# Genome-Wide Association Studies of Photosynthetic Traits Related to Phosphorus Efficiency in Soybean

**DOI:** 10.3389/fpls.2018.01226

**Published:** 2018-08-28

**Authors:** Haiyan Lü, Yuming Yang, Haiwang Li, Qijia Liu, Jianjun Zhang, Junyi Yin, Shanshan Chu, Xiangqian Zhang, Kaiye Yu, Lingling Lv, Xi Chen, Dan Zhang

**Affiliations:** ^1^Collaborative Innovation Center of Henan Grain Crops, Henan Agricultural University, Zhengzhou, China; ^2^National Center for Soybean Improvement, National Key Laboratory of Crop Genetics and Germplasm Enhancement, Nanjing Agricultural University, Nanjing, China; ^3^College of Food Science and Technology, Henan University of Technology, Zhengzhou, China

**Keywords:** soybean, photosynthesis-related traits, phosphorus efficiency, multi-locus GWAS, QTNs, candidate gene, mrMLM

## Abstract

Photosynthesis is the basis of plant growth and development, and is seriously affected by low phosphorus (P) stress. However, few studies have reported for the genetic foundation of photosynthetic response to low P stress in soybean. To address this issue, 219 soybean accessions were genotyped by 292,035 high-quality single nucleotide polymorphisms (SNPs) and phenotyped under normal and low P conditions in 2015 and 2016. These datasets were used to identify quantitative trait nucleotides (QTNs) for photosynthesis-related traits using mrMLM, ISIS EM-BLASSO, pLARmEB, FASTmrMLM, FASTmrEMMA, and pKWmEB methods. As a result, 159 QTNs within 31 genomic regions were found to be associated with four photosynthesis-related traits under different P stress conditions. Among the 31 associated regions, five (*q7-2, q8-1, q9, q13-1*, and *q20-2*) were detected commonly under both normal and low P conditions, indicating the insensitivity of these candidate genes to low P stress; five were detected only under normal P condition, indicating the sensitivity of these candidate genes to low P stress; six were detected only under low P condition, indicating the tolerantness of these candidate genes to low P stress; 20 were reported in previous studies. Around the 159 QTNs, 52 candidate genes were mined. These results provide the important information for marker-assisted breeding in soybean and further reveal the basis for the application of P tolerance to photosynthetic capacity.

## Introduction

Phosphorus (P) is one of the main factors for plant growth because of its influence on cellular phosphorylation events and the synthesis of DNA and RNA (Johnston et al., 2000; Khan et al., 2009; Zhang et al., 2014b; Li et al., 2016). Nevertheless, the availability of P in soil is limited owing to the formation of organic P complexes and the fixation of P by aluminum and ferrum oxides (Vance et al., 2003; Wang et al., 2010). In the past decade, enormous efforts have been made in the dissection of the genetic mechanisms for soybean P efficiency by evaluating factors such as P concentration, root architecture (Ao et al., 2010), biomass (Li et al., 2005), and phosphatase activity (Zhang et al., 2009). Although a series of quantitative trait loci (QTLs) across all 20 chromosomes on the genome have been found to be associated with P efficiency in soybean (SoyBase, https://soybase.org), QTLs underlying photosynthetic response to low P stress have rarely been studied.

Plant productivity relies on photosynthesis, which is sensitive to low P stress (Veneklaas et al., 2012). A number of QTLs associated with photosynthesis-related traits have been detected (Yin et al., 2010a,b; Hao et al., 2012). However, the situation under the low P stress has not been considered. Recently, linkage mapping studies showed a significant genetic relationship between P efficiency and photosynthesis-related traits, such as net photosynthetic rate and transpiration rate (Li et al., 2016). In soybean, however, both the P efficiency and photosynthesis-related traits are complex quantitative traits controlled by polygenes, and most of them are genotype-specific and environment-sensitive. So far, no pleiotropic QTL for the two traits have been reported, mainly because of the relatively low mapping resolution and smaller allele effect sizes.

More recently, genome-wide association study (GWAS) has a great advantage in the dissection of genetic basis of complex traits over linkage analysis: GWAS leverages the greater number of historical recombination events, a greater number of alleles, and broader genetic variation (Yu and Buckler, 2006). Up to now this approach was widely used in multiple crops, for instance, in rice (Huang et al., 2012), soybean (Zhang et al., 2014a,b), maize (Mao et al., 2015; Wang et al., 2016c), and *Arabidopsis thaliana* (van Rooijen et al., 2017).

The most popular method for GWAS is mixed linear model (MLM) method (Zhang et al., 2005; Yu and Buckler, 2006). In the past decade, many MLM-based methods have been proposed to improve computational efficiency, such as CMLM (Zhang et al., 2010) and ECMLM (Li et al., 2014). However, these models are one-dimensional genome scan, which need the correction for multiple tests. The typical Bonferroni correction is often too conservative to identify many important loci with small effects. To address this problem, Wang et al. (2016b) proposed a multi-locus random-SNP-effect mixed linear model (mrMLM) method without Bonferroni correction. And then, a series of multi-locus GWAS methods have been proposed, such as ISIS EM-BLASSO (Tamba et al., 2017), pLARmEB (Zhang et al., 2017), FASTmrEMMA (Wen et al., 2018), FASTmrMLM (Tamba and Zhang, 2018), and pKWmEB (Ren et al., 2018). These methods not only improve the power and accuracy of GWAS but also identify the small-effect quantitative trait nucleotides (QTNs).

To reveal the genetic basis of photosynthetic response to low P stress in soybean, in this study, four photosynthesis-related traits under two P levels were measured for seedling plants in hydroponics across two environments, 219 soybean accessions were genotyped by 292,035 high-quality SNPs from NJAU 355 K Soy SNP array described by Wang et al. (2016a), and the two datasets were used to conduct GWAS for the above four traits. Because of the relatively smaller allelic effects, multi-locus GWAS methods as mentioned above, rather than common GWAS methods based on single marker analysis with a fixed-SNP-effect MLM, were adopted in this study.

Our objectives were: (i) to estimate the genetic variance and heritability of four photosynthesis-related traits under different P conditions; (ii) to investigate the correlations among the four traits under different P levels; (iii) to detect QTNs associated with the above four traits; and (iv) to predict their candidate genes.

## Materials and methods

### Plant materials and hydroponics experiments

The population for GWAS was comprised of 219 soybean accessions (including 195 landraces and 24 elite varieties) derived from 26 provinces within six ecological regions in China (latitude 53 to 24°N and longitude 134 to 97°E; Wang and Gai, 2002). The 219 soybean accessions were grown hydroponically and measured by two independent experiments in 2015 and 2016 (E1 and E2). Hydroponics experiments and phenotyping were conducted as previously described by Li et al. (2016). The controlled conditions of hydroponics was 28/20°C day/night temperature and 10 h light/14 h dark photoperiod in artificial climate chambers. The surfaces of the seeds were sterilized with chlorine, and then, the seeds were sprouted in sterile vermiculite. Next, regular soybean seedlings, whose cotyledons were expanded completely, were selected. Then, the selected seedlings were moved into modified one-half Hoagland's nutrient solution supplemented with 500 μM P (normal P, KH2PO4) for 3 days. Finally, one half of the seedlings were transferred into modified one-half Hoagland's nutrient solution supplemented with 5 μM P (low P) for 14 days, and the other half remained in the normal P condition as controls.

The photosynthesis-related traits assessed were net photosynthetic rate (Pn, μmol·m^2^·s^−1^), transpiration rate (Tr, g·m^2^·h^−1^), stomatal conductance (Co, mmol·m^−2^· s^−1^), and intercellular carbon dioxide concentration (Ci, μL·L^−1^) under different P conditions (normal P, low P, and the ratio of low/normal P were abbreviated as NP, LP, and L/NP, respectively) in 2015 (E1) and 2016 (E2). A LI-6400 portable photosynthesis system was used to measure the above four traits (Li Cor Inc., Lincoln, NE, USA). The phenotyping used the upper third leaf of three plants, and three replicates were measured per plant. All the traits were measured at the second trifoliolate stage. A total of 12 characteristics were analyzed in this paper: PnNP, PnLP, PnL/NP represent the net photosynthetic rates under normal P, low P, and the ratios of low/normal P, respectively; and TrNP, TrLP, TrL/NP, CoNP, CoLP, CoL/NP, CiNP, CiLP, CiL/NP represent the transpiration rates, stomatal conductance and intercellular carbon dioxide concentrations under normal P, low P, and the ratios of low/normal P, respectively.

### Genotyping and statistical analysis of the phenotypes

Two hundred and nineteen soybean accessions were genotyped by 292,035 SNPs derived from NJAU 355 K Soy SNP array described by Wang et al. (2016a). In other words, there was one SNP per 3.3 kb along the 20 soybean chromosomes. In the present study, SNPs with minor allele frequency (MAF) < 0.05 were deleted. Based on this rule, a total of 201,994 SNPs were used for the GWAS.

The ANOVA of the phenotypic data was carried out using the PROC GLM of SAS version 9.2 (SAS Institute, Cary, NC, USA). Genotype and environment were treated as fixed and random, respectively. The broad-sense heritability (*h*^2^) was calculated as: $h^{2}=\sigma_{g}^{2}/\sigma_{p}^{2}$, where $\sigma_{g}^{2}$ is the genotypic variance, $\sigma_{p}^{2}$ is the phenotypic variance. SPSS Statistics 19.0 (SPSS, Inc., Chicago, IL, USA) was used to analyze the correlation coefficients among the four photosynthesis-related traits under different P conditions in the soybean.

### Genome-wide association studies and prediction of candidate genes

Population structure of the 219 soybean accessions each with 201,994 SNPs was calculated using the STRUCTURE package (Pritchard et al., 2009). The relative kinship (K matrix) between a pair of accessions was calculated using the R package mrMLM. GWAS was performed by the R package mrMLM with six multi-locus GWAS methods: mrMLM (Wang et al., 2016b), ISIS EM-BLASSO (Tamba et al., 2017), pLARmEB (Zhang et al., 2017), FASTmrEMMA (Wen et al., 2018), FASTmrMLM (Tamba and Zhang, 2018), and pKWmEB (Ren et al., 2018). In order to get more accurate candidate genes, markers that met the criterion of LOD score ≥5 were considered to be significantly associated with the traits.

To mine the candidate genes related to soybean photosynthesis response to low P stress, the predicted genes around significantly associated QTNs were identified based on the annotation in the soybean reference genome (Wm82.a2.v1) in Phytozome v10.3 (http://phytozome.net). Then, the genes with known function annotations underlying soybean photosynthesis-related traits under different P conditions were selected as candidate genes. In addition, we also selected the previously reported QTLs from soybase (https://soybase.org) in the associated genomic regions.

## Results

### Phenotype for photosynthesis-related traits

**Figure 1:**
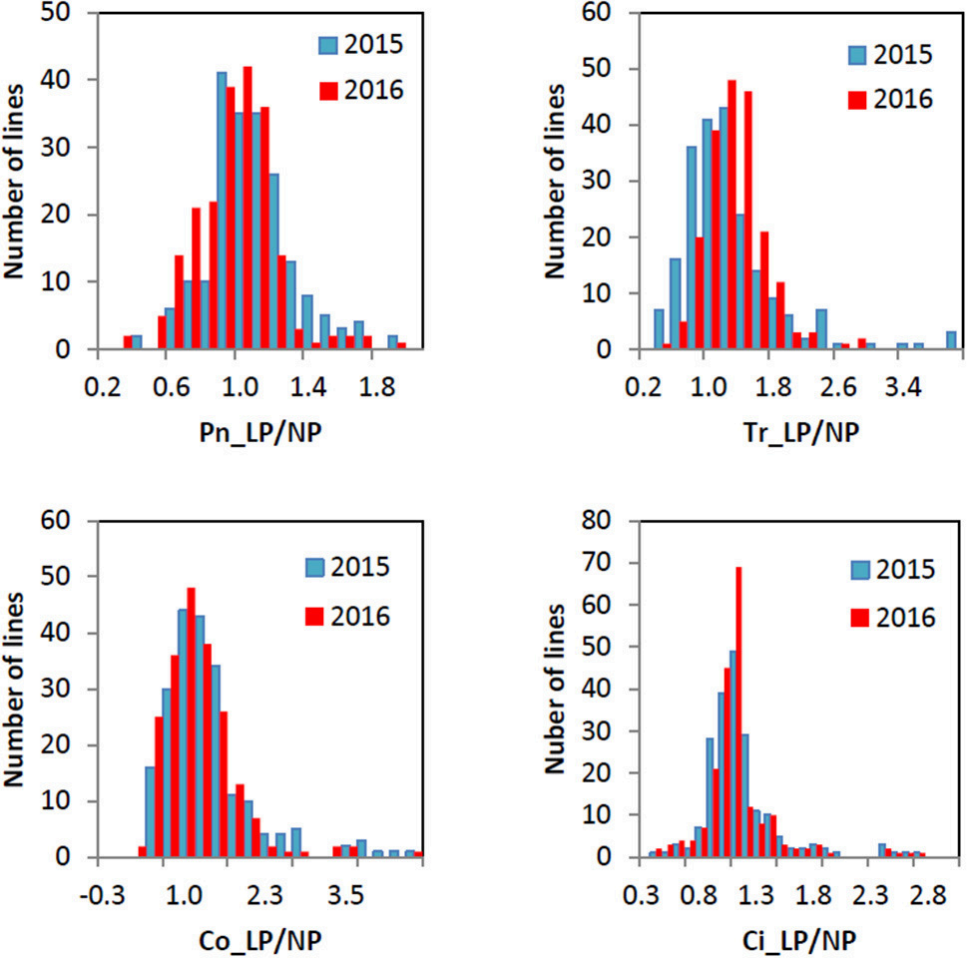
Histogram of the frequency distributions for the four photosynthesis-related traits in soybean under L/NP condition in 2015 and 2016.

All the four traits under different P conditions showed approximately normal distributions (Figure 1 and Figure S1). However, the four traits under the L/NP condition were far away from normal distributions, indicating the existence of major QTNs. The coefficients of variation for the four traits under different P conditions ranged from 13.99~69.22% (Table 1). The analysis of variance showed the significant differences for the four traits between genotypes and between environments. The last two results indicated that it is suitable for this population to conduct multi-locus GWAS.

**Table 1:** Descriptive statistical results for photosynthesis-related traits of soybean under different P conditions.

**P level^a^**	**Trait**	**Year**	**Mean**	**Stdev**	**Skewness**	**Kurtosis**	**Minimum**	**Maximum**	**CV(%)^b^**	**Genotype**	**Environment**	***h*^2^(%)^c^**
NP	Pn	2015	15.66	6.87	0.83	−0.25	3.06	34.38	43.8	^**^	^**^	81.32
		2016	19.00	7.54	0.51	−0.97	4.89	36.17	39.68			
	Tr	2015	3.11	1.61	1.58	3.62	0.85	10.67	51.82	^**^	^**^	72.33
		2016	4.79	2.51	0.46	−0.70	1.01	10.73	52.45			
	Co	2015	0.15	0.08	0.78	0.28	0.03	0.39	53.24	^**^	^**^	78.66
		2016	0.20	0.12	0.80	0.39	0.04	0.67	58.07			
	Ci	2015	391.97	138.66	1.54	3.11	131.02	972.15	35.37	^**^	^**^	74.45
		2016	301.55	43.09	0.29	2.68	131.02	444.70	14.29			
LP	Pn	2015	14.90	6.56	0.72	−0.76	4.10	31.79	44.0	^**^	^**^	76.69
		2016	16.99	8.48	0.50	−0.99	2.64	36.50	49.91			
	Tr	2015	3.14	1.84	1.95	5.08	0.80	11.78	58.76	^**^	^**^	75.00
		2016	4.53	2.73	0.84	−0.01	0.43	12.56	60.23			
	Co	2015	0.16	0.10	1.44	2.17	0.03	0.53	63.43	^**^	^**^	80.83
		2016	0.19	0.13	1.19	1.00	0.02	0.67	69.14			
	Ci	2015	410.08	139.91	1.76	3.71	130.76	954.63	34.12	^**^	^**^	69.79
		2016	312.03	43.65	0.68	0.76	213.21	457.05	13.99			
L/NP	Pn	2015	1.02	0.29	2.41	15.05	0.35	3.21	28.7	^**^	^**^	47.58
		2016	0.89	0.23	0.59	2.31	0.25	1.88	26.25			
	Tr	2015	1.16	0.64	2.31	7.30	0.26	4.39	55.4	^**^	^**^	63.33
		2016	1.03	0.49	2.19	7.55	0.14	3.59	47.26			
	Co	2015	1.29	0.89	2.49	7.47	0.27	5.75	69.2	^**^	^**^	85.14
		2016	1.09	0.72	3.09	14.28	0.09	5.75	66.19			
	Ci	2015	1.14	0.44	3.14	13.25	0.37	3.82	38.9	^**^	^**^	91.73
		2016	1.12	0.67	3.21	13.97	0.32	3.82	59.62			

** *: the 0.01 level of significance*.

a *P level: NP, LP, and L/NP represent the traits under normal P, low P, and the ratio of low and normal P, respectively*.

b *Coefficient of variation*.

c *broad-sense heritability*.

To investigate the correlation among the four photosynthesis-related traits, simple correlations were calculated based on the average values of the two experiments (Table 2 and Table S2). The results showed that Co was very significantly and positively correlated with Tr [*r* = 0.886 (NP) or 0.924 (LP)]; Ci was very significantly and positively correlated with Pn [*r* = 0.394 (NP) or 0.500 (LP)]; Pn was significantly and negatively correlated with Tr and Co (*r* = −0.100 and −0.108), respectively, under normal P condition; Tr was significantly and negatively correlated with Ci (*r* = −0.167) under normal P condition (Table 2).

**Table 2:** Correlations between four photosynthesis-related traits under different P conditions.

**LP/NP**	**Pn**	**Tr**	**Co**	**Ci**
Pn	1.000	−0.100^*^	−0.108^*^	0.394^**^
Tr	0.016	1.000	0.886^**^	−0.167^**^
Co	0.015	0.924^**^	1.000	0.023
Ci	0.500^**^	−0.096	0.073	1.000

*Pearson correlation coefficients under NP and LP conditions were listed above and below the diagonal, respectively*.

* and

** *: the 0.05 and 0.01 levels of significance, respectively*.

### Multi-locus genome-wide association studies for photosynthesis-related traits

**Figure 2:**
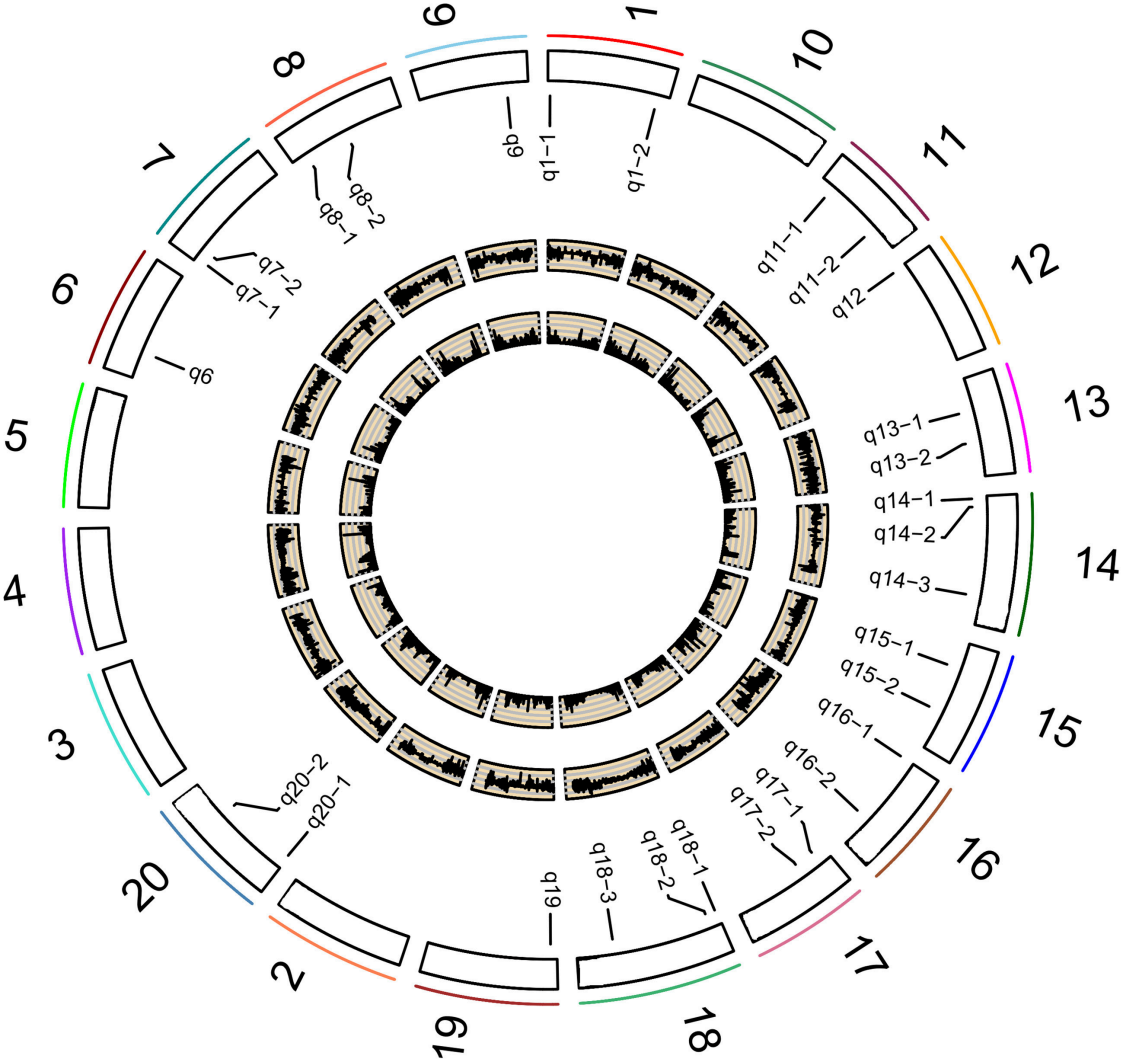
Soybean chromosomes and QTLs for the studied traits under different P conditions. The outside/inside wheat-colored circle indicates the LOD/ *r*^2^ value curve for the studied traits across environments. The outermost circle indicates the 20 soybean chromosomes; QTLs for the studied traits under different P conditions.

A total of 201,994 SNPs were selected with MAF ≥ 0.05 from 292,053 high-quality SNPs. The selected SNPs were used to determine the number of sub-populations (*k*) using the software STRUCTURE. As a result, the *k*-value was 3. The above information along with four photosynthesis-related traits under different P conditions (NP, LP, and L/NP) in 2015 (E1) and 2016 (E2) was used to conduct multi-locus GWAS using package mrMLM. For all the traits, QTNs within approximately 5 Mb or less were viewed as caused by one common gene (Visscher et al., 1996; Öckinger et al., 2006; Swanson-Wagner et al., 2009; Wang et al., 2012). As a result, a total of 31 associated regions comprised of 159 QTNs across all the 20 soybean chromosomes, except the 2, 3, 4, 5, and 10 chromosomes, were significantly associated with the related traits at the critical LOD ≥5 (Table 3 and Figure 2). All the 31 associated regions were identified by at least three methods. The full list of significant QTNs from the six multi-locus GWAS methods is presented in Table S2. Among the 159 QTNs, the numbers of QTNs detected under NP, LP, and L/NP conditions were 59, 64, and 66, respectively; while the numbers of QTNs associated with Co, Tr, Ci, and Pn were 56, 54, 35, and 31, respectively (Table S2).

**Table 3:** Details of loci associated with photosynthesis-related traits via multi-locus GWAS in soybean.

**Region associated^a^**	**Chr.^b^**	**SNP associated^c^**	**Pos. (bp)^d^**	**No.^e^**	**LOD**	**r^2^ (%)^f^**	**Position intervals (bp)**	**Method^g^**	**Trait-year-treatment^h^**
q1-1	1	AX-93961332	951461	5	6.32	3.35	951461–1344144	1, 3, 5	TrLP_E1, CoLP_E1, TrL/NP_E2, CoL/NP_E1
q1-2	1	AX-93675249	50496989	1	11.37	8.83	50496989	1, 2, 3	PnLP_E2, PnNP_E2
***q6***	**6**	**AX-93728015**	**12548698**	**7**	**9.18**	**11.51**	**12516126–12966564**	**1, 2, 3, 4, 5**	**TrLP_E1, CoLP_E2, TrNP_E2, CoNP_E2, TrL/NP_E2, CoL/NP_E2**
q7-1	7	AX-93926489	3402895	4	7.68	11.06	1787229– 3402895	1, 3, 5	CiLP_E2, CoL/NP_E1, CiL/NP_E1
***q7-2***	**7**	**AX-93741303**	**9520676**	**7**	**6.55**	**4.46**	**6578651– 9520676**	**1, 2, 3, 5**	**PnLP_E2, CiLP_E2, TrNP_E2, CoNP_E2, TrL/NP_E1, CoL/NP_E1, CoL/NP_E2**
***q8-1***	**8**	**AX-93753054**	**11620180**	**7**	**7.82**	**6.41**	**6955757–11620180**	**1, 2, 3, 4, 5**	**PnLP_E2, TrLP_E1, CoLP_E1, PnNP_E1, CoNP_E1, CoNP_E2, TrL/NP_E1,**
q8-2	8	AX-93929582	27620272	1	6.54	3.19	27620272	1, 2, 3, 4	PnLP_E2, PnNP_E2
***q8-3***	**8**	**AX-93759645**	**43743823**	**6**	**9.87**	**9.64**	**41226360–46081608**	**1, 2, 4, 5, 6**	**PnNP_E1, PnNP_E2, TrNP_E2, CoNP_E2, CiNP_E2, TrL/NP_E1, CoL/NP_E1, CiL/NP_E2**
***q9***	**9**	**AX-94066868**	**40240035**	**6**	**12.20**	**13.77**	**40188126–42709534**	**1, 2, 3, 4, 5**	**TrLP_E2, CoLP_E2, TrNP_E1, CoNP_E2, CiNP_E1, TrL/NP_E1, TrL/NP_E2, CiNP_E2**
q11-1	11	AX-94084631	6262749	4	8.13	7.09	6262749–9894114	2, 4, 5	PnNP_E1, PnNP_E2, CoL/NP_E2, CiL/NP_E1
q11-2	11	AX-94091690	32599188	4	6.92	9.87	32540598–34020885	2, 3, 4, 5	TrNP_E1, CoNP_E1, CiNP_E2, CoL/NP_E2, CiL/NP_E2
q12	12	AX-93796430	1697221	2	8.18	7.24	613090–1697221	1, 3, 4, 5	CiNP_E2, CiL/NP_E2
***q13-1***	**13**	**AX-94104819**	**18590366**	**4**	**28.95**	**6.31**	**15071765–18590366**	**1, 2, 3,**	**TrLP_E1, CoLP_E1, CiLP_E1, PnNP_E1, CiNP_E1, CoNP_E1, CoL/NP_E2**
***q13-2***	**13**	**AX-94287210**	**31003637**	**5**	**10.38**	**1.28**	**29481274–31003637**	**1, 3, 4, 5**	**PnNP_E2, TrNP_E2, PnL/NP_E1, TrL/NP_E1, CiL/NP_E1, CiL/NP_E2**
q14-1	14	AX-93820315	1059966	5	9.24	7.03	1059966–2165525	1, 2, 4, 5, 6	PnLP_E2, PnNP_E2, TrL/NP_E2, CoL/NP_E2, CiL/NP_E2
q14-2	14	AX-94288085	4897088	4	21.44	7.25	4897088–7755174	1, 3, 4, 5	CoLP_E2, CiLP_E2, TrNP_E2, CoNP_E2, TrL/NP_E2
***q14-3***	**14**	**AX-94129538**	**47514182**	**7**	**7.27**	**5.19**	**46008634–47723841**	**2, 3, 4, 5**	**PnLP_E1, CoLP_E1, PnL/NP_E1, PnL/NP_E2, TrL/NP_E1, CiL/NP_E2**
q15-1	15	AX-94134672	12611721	3	6.26	2.32	12227172–12611721	1, 3, 4	CoLP_E2, CoNP_E1, CoNP_E2, PnL/NP_E1
q15-2	15	AX-93841986	35259050	2	7.39	5.01	33719705–35259050	1, 2, 4, 5	PnLP_E2, TrLP_E2, CoLP_E2
q16-1	16	AX-93946322	179538	5	6.73	5.10	147918–2435036	1, 2, 4, 5	TrNP_E2, CoNP_E1, CoNP_E2, CiNP_E2
q16-2	16	AX-93947209	35210924	7	10.99	7.30	32784699–37003959	1, 2, 4, 5	TrLP_E1, TrLP_E2, CoLP_E1, CoLP_E2, CoL/NP_E2
q17-1	17	AX-94155900	5718961	4	11.38	9.51	5718961–5761052	2, 3, 5	CoLP_E2, PnNP_E1, TrNP_E2, CiL/NP_E1
q17-2	17	AX-94159333	15188572	2	7.19	8.91	15188572–15492731	2, 3, 4, 5	CiLP_E1, CiNP_E1
q17-3	17	AX-93866265	37701731	5	8.32	9.24	37615577–39224452	1, 2, 3, 4	TrLP_E1, PnNP_E1, TrNP_E2, TrL/NP_E2, CoL/NP_E2
***q18-1***	**18**	**AX-94166205**	**2982489**	**7**	**15.10**	**12.61**	**674420–3925002**	**2, 3, 4, 5**	**PnLP_E1, TrLP_E1, CiLP_E1, CiLP_E2, CiNP_E1, TrL/NP_E2**
***q18-2***	**18**	**AX-93871255**	**9956907**	**6**	**9.86**	**15.88**	**5443584-9956907**	**1, 2, 3, 4, 5**	**PnLP_E1, TrLP_E1, TrLP_E2, CoLP_E2, PnNP_E2, CoL/NP_E1**
***q18-3***	**18**	**AX-93883305**	**53037663**	**15**	**13.24**	**10.91**	**50663235- 55727445**	**1, 2, 3, 5, 6**	**PnLP_E2, TrLP_E2, CoLP_E1, PnNP_E2, CoNP_E1, CoNP_E2, PnL/NP_E1, TrL/NP_E1, CoL/NP_E1, CiL/NP_E1, CiL/NP_E2**
q19	19	AX-93886366	2991135	4	8.17	9.02	2991135- 3374702	1, 4, 5, 6	TrLP_E1, TrLP_E2, CoLP_E1, CoLP_E2
***q20-1***	**20**	**AX-94197533**	**1364621**	**6**	**8.20**	**6.22**	**455608-3145850**	**2, 3, 4, 5**	**CoLP_E1, PnL/NP_E1, TrL/NP_E1, TrL/NP_E2, CoL/NP_E1, CiL/NP_E2**
***q20-2***	**20**	**AX-93956837**	**35089898**	**10**	**11.48**	**16.82**	**35089898-39987379**	**1, 2, 3, 5, 6**	**PnLP_E1, TrLP_E1, CoLP_E1, CiLP_E1, PnNP_E1, CoNP_E1, PnL/NP_E2, TrL/NP_E1, TrL/NP_E2, CoL/NP_E2**
q20-3	20	AX-93910666	45122261	4	8.91	7.38	44049520- 46908248	1, 2, 3, 4	TrNP_E1, CoNP_E1, CiNP_E2, CoL/NP_E2

a *QTN named by chromosome*.

b *Chromosome*.

c *QTNs that were significantly associated with the trait*.

d *QTN position (bp) on soybean genome assembly Glycine max Wm82.a1.v1.1 (www.phytozome.net)*.

e *The number of significant QTNs detected in the region*.

f *The proportion of phenotypic variance explained by each QTN*.

g *The mrMLM, pKWmEB, pLARmEB, FASTmrMLM, ISIS EM-BLASSO, and FASTmrEMMA were marked from 1 to 6, respectively*.

h *The trait-year-treatment combination of QTN, for example, Pn, net photosynthetic rate; Tr, transpiration rate; Ci, intercellular carbon dioxide concentration; and Co, stomatal conductance; followed by the treatments and environments. NP, LP and L/NP denote normal-P, low-P and the ratio of low/normal P condition, respectively. E1 and E2 denote 2015 and 2016, respectively*.

*The bold values denote major QTL*.

Most of the 31 associated regions were detected under both NP and LP conditions, including the QTNs on chromosomes 1, 6, 7, 8, 9, 13, 14, 15, 18, and 20. Five of them, *q7-2, q8-1, q9, q13-1*, and *q20-2* were more representative than others. *q13-1* was associated with the four photosynthesis-related traits, Pn, Tr, Co, and Ci. *q8-1* was associated with Tr, Co, and Pn. *q9* was associated with Tr, Co, and Ci. Additionally, *q8-1* and *q9* were associated with Tr under the L/NP conditions. *q7-2* was associated with Pn and Ci under LP conditions, while it was also associated with Tr and Co under NP conditions. *q20-2* was associated with Pn and Co under both NP and LP conditions, while it was also associated with Tr and Ci under LP conditions. There were some regions that were uniquely associated with one trait. For example, *q17-2* was associated only with Ci, while *q1-2* and *q8-2* were associated only with Pn, and both loci contain only one QTN. These QTNs probably contribute to the genetic basis of photosynthesis and are probably not significantly influenced by low P stress.

In addition, there were several QTNs identified uniquely under NP or LP conditions and, therefore, they were considered as NP-specific or LP-specific QTNs. For example, *q8-3, q11-2, q13-2, q16-1*, and *q20-3* on chromosomes 8, 11, 13, 16, and 20, respectively, were detected only under NP conditions. In contrast, several LP-specific QTNs, *q1-1, q7-1, q14-3, q15-2, q16-2*, and *q19* on chromosomes 1, 7, 14, 15, 16, and 19, respectively, were detected only under LP level, indicating that the genes underlying these QTNs may be more likely to be affected by low P stress. Moreover, most of the 31 loci were detected under L/NP conditions, and the most representative QTNs were *q18-3* and *q20-1*, which were associated with all four photosynthesis-related traits under the L/NP conditions. Further research of these P condition-specific QTNs may supply more understanding to the genetic basis of P tolerance to photosynthetic capacity.

On the other hand, 13 of the 31 associated regions were repeatedly detected more than 6 times across treatments, traits or years, which are major QTNs (Figure 2; Table 3). These QTNs could be used to assess the effect of low P stress on photosynthesis in further analysis. As shown in Table 3, 13 QTNs (*q6, q7-2, q8-1, q8-3, q9, q13-1, q13-2, q14-3, q18-1, q18-2, q18-3, q20-1*, and *q20-2*) were mapped on chromosomes 6, 7, 8, 9, 13, 14, 18, and 20. In addition, comparative analyses showed that eight major QTNs (*q7-2, q8-1, q9, q13-2, q14-3, q18-2, q18-3*, and *q20-1*) were co-localized with the QTLs identified in previous reports (Zhang et al., 2009, 2016; Li et al., 2016), including the QTL harboring the P efficiency-related gene, *GmACP1* (Zhang et al., 2014b). These eight QTNs most likely play important roles for P efficiency in soybean. For instance, the major QTN *q8-1*, where the acid phosphatase encoding gene *GmACP1* is located and underlying variation in Pn, Tr, and Co, was stably detected across traits and environments. The co-localization of *GmACP1* with *q8-1* demonstrates the high accuracy of the GWAS results in this study.

### Prediction and preliminary validation of candidate genes

Although it is not easy to compare the results in different studies with different genetic maps, we determined whether the 31 associated regions in the present study were situated at or near the same position as previously identified QTLs by comparing the chromosomal locations of these QTLs (https://soybase.org). Twenty of the 31 regions were reported in previous studies (Table S3), and some of them were associated with leaflet-related traits (Yamanaka et al., 2001; Jun et al., 2014; Shim et al., 2015), such as *q7-2, q14-2, q18-2*, and *q20-2*, which could be closely related with photosynthesis.

To identify candidate genes affecting each trait, we re-investigated the 159 QTNs detected in our study based on the annotation of the soybean reference genome W82.a2.v1. As a result, 52 annotated genes were found and listed in Table S4. Most of them were previously associated with P efficiency. For example, the gene cluster within the *q8-1* region on chromosome 8 (*Glyma.08G114800, Glyma.08G115400, Glyma.08G123200, Glyma.08G129200, Glyma.08G150800*) was near the key P efficiency-related gene, *GmACP1* (Zhang et al., 2014b). Another gene cluster within the *q13-2* region on chromosome 13 (*Glyma.13G181600, Glyma.13G192100, Glyma.13G194500*, and *Glyma.13G196600*) was near the protein kinase gene, *Glyma.13G161900* (Zhang et al., 2016). In particular, the gene *Glyma.13G196600*, encoding NADPH: quinine oxidoreductase, might participate in the metabolic processes involving phosphate and photosynthesis.

The major gene cluster of *q18-1* on chromosome 18 has three annotated genes in the region encoding DNA polymerase alpha 2 (*Glyma.18G009300*), anaphase-promoting complex/cyclosome 2 (*Glyma.18G036900*), and ALWAYS EARLY4 (*Glyma.18G040400*), which is near a rubisco activase gene *Glyma.18G036400* (Li et al., 2016); these could have significant effects on the regulation of photosynthetic capacity in the soybean. In addition, there was also a single annotated gene that had been reported previously. *Glyma.12G023100*, within the *q12* region on chromosome 12, encodes a Transmembrane amino acid transporter family protein, which is physically close to ribulose-bisphosphate carboxylases gene *Glyma.12G061600* (Li et al., 2016).

## Discussion

### Comparison of six multi-locus GWAS methods

With the development of advanced genomic sequencing technologies, GWAS has become a widely used method and is popular for the genetic dissection of variation in complex traits. While most complex traits are dominated by major genes plus polygenes, the common GWAS using a one-dimensional scanning model might not be able to detect associations with the variation of polygenes because of the limitation of the model. A better alternative is the multi-locus model GWAS (Wang et al., 2016b). In the present study, six multi-locus GWAS methods were used, and a total of 159 QTNs were found to be associated with the four photosynthesis-related traits under different P conditions (Table 4 and Table S2). Furthermore, 41 of the 159 QTNs were detected by at least two methods and all the 31 associated regions were detected by at least three methods. In comparing the six multi-locus GWAS methods, we found that only nine QTNs had been detected by FASTmrEMMA, while more than 40 QTNs were detected by each of the other five methods.

**Table 4:** Summary of six multi-locus GWAS analysis for the four traits.

**Case**	**mrMLM**	**pKWmEB**	**pLARmEB**	**FASTmrMLM**	**ISIS EM-BLASSO**	**FASTmrEMMA**
No. QTN^a^	46	55	52	44	43	9
No. region^b^	24	24	24	23	25	5
LOD	5.02~11.48	5.11~13.24	5.12~28.95	5.03~10.99	5.08~11.38	5.02~8.26
*r*^2^ (%)^c^	3.35~16.82	3.49~12.27	0.01~13.77	0.62~9.82	2.11~16.04	5.06~9.14

a *The number of detected QTNs*.

b *The number of associated regions*.

c *The proportion of phenotypic variance explained by each QTN (%)*.

The maximum LOD scores were more than 10 except for those from FASTmrEMMA, which was 8.26, smaller than the other five methods. The maximum LOD score of pLARmEB (28.95) was significantly larger than the LOD scores from the other methods. Moreover, the minimum *r*^2^ (%) was 0.01 from pLARmEB, which may be meaningless. Meanwhile, the minimum *r*^2^ (%) from FASTmrEMMA was 5.06, which was significantly higher than those from the other methods, meaning that FASTmrEMMA might detect major QTNs with the larger effects. This outcome explains why there were fewer associated QTNs from FASTmrEMMA than from the other five methods.

### Novel QTNs and potential candidate genes of interest

Among the 13 major QTNs, five (*q6, q8-3, q13-1, q18-1*, and *q20-1*), which have not been reported in previous studies, were considered as novel QTNs for photosynthesis response to low P stress. It is worth noting that *q20-1* was associated with all four photosynthesis-related traits under the L/NP conditions. Thus *q20-1* might represent another important novel QTN related to Photosynthesis. In addition, two annotated genes within the *q20-1* region encoding a Mitochondrial substrate carrier family protein (*Glyma.20G004600*) and a Cyclophilin-like peptidyl-prolyl cis-trans isomerase family protein (*Glyma.20G005600*) were found in our study. If possible, more research on these genes might reveal their genetic mechanisms in future.

Another major QTN, *q13-1*, was associated with the four photosynthesis-related traits under both NP and LP conditions. This QTN was also reported previously for seed methionine content and seed cysteine content (Panthee et al., 2006a,b). Furthermore, one annotated gene *Glyma.13G053400*, within the *q13-1* region on chromosome 13, which encodes a Mitochondrial substrate carrier family protein, was listed in Table S4. Thus, this QTN could be a promising candidate locus for further study of low P stress on photosynthetic efficiency.

Some annotated genes weren't reported previously to be associated with phosphate and photosynthetic metabolic processes. For instance, one gene *Glyma.14G029100*, within the *q14-1* region on chromosome 14, encodes sucrose phosphate synthase 3F. Two annotated genes encoding a phosphate transporter (*Glyma.11G087800*) and a phospholipase (*Glyma.11G230100*) might be involved in the metabolic process of phosphate and photosynthesis.

Based on 292,035 high-quality SNPs in 219 soybean accessions, 159 QTNs within 31 regions were identified to be associated with four photosynthesis-related traits under different P conditions. Importantly, genetic improvement simultaneously for phosphorus efficiency and photosynthesis in soybean might be carried out by selecting for a single large-effect QTN. The associated regions and candidate genes detected in the present study could be further tested for marker-assisted breeding of soybean varieties for the application of P tolerance to photosynthetic capacity.

## Author contributions

DZ and HLü conceived and designed the experiments. YY, HLi, SC, XZ, KY, LL, XC, and WW performed the experiments. HLü, JZ, JY, and QL performed data analyses. HLü and DZ wrote the manuscript. All authors have read and approved the final version of the manuscript.

### Conflict of interest statement

The authors declare that the research was conducted in the absence of any commercial or financial relationships that could be construed as a potential conflict of interest. The reviewer JZ declared a shared affiliation, though no other collaboration, with one of the authors YY to the handling Editor.

## Supplementary material

Figure S1 Histogram of the frequency distributions for the four photosynthesis-related traits of soybean under NP and LP conditions in 2015 and 2016.

Table S1 Correlation coefficients among the four photosynthesis-related traits under different P conditions.

Table S2 The detailed list of QTNs for photosynthesis-related traits under different P conditions across two environments using six multi-locus GWAS methods.

Table S3 The overlapped QTLs between present and previous studies.

Table S4 Predicted candidate genes associated with photosynthesis-related traits under different *P* conditions.
