# Supplementary figures and images for: Genome-Wide Association Studies of Photosynthetic Traits Related to Phosphorus Efficiency in Soybean
Source: Front Plant Sci. 2018 Aug 28;9:1226. doi: 10.3389/fpls.2018.01226 (PMC6122521; doi:10.3389/fpls.2018.01226)

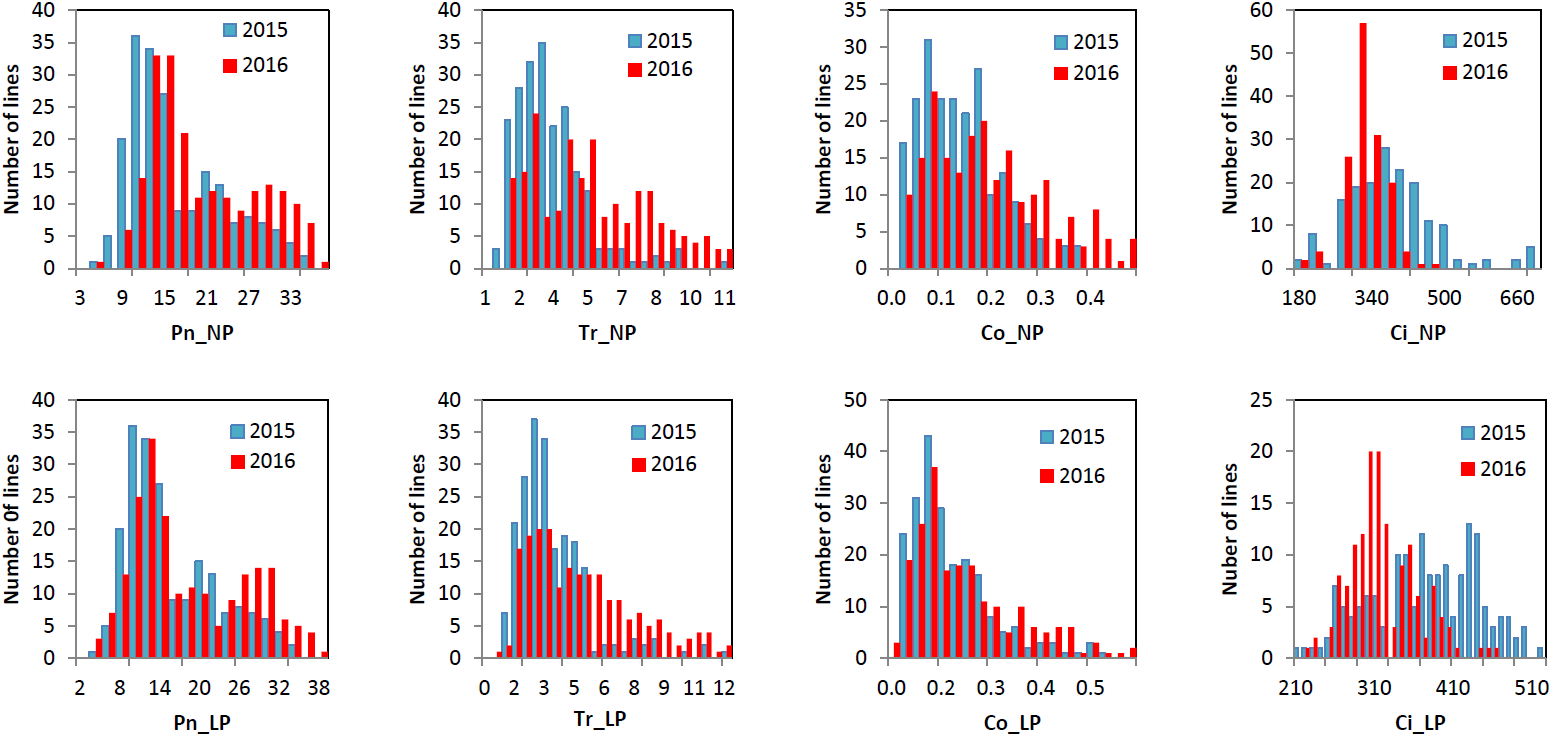

Supplement: Figure S1 — Histogram of the frequency distributions for the four photosynthesis-related traits of soybean under NP and LP conditions in 2015 and 2016. [file Image_1.TIF]
